# Performance of *Leishmania* PFR1 recombinant antigen in serological diagnosis of asymptomatic canine leishmaniosis by ELISA

**DOI:** 10.1186/s12917-017-1224-z

**Published:** 2017-10-23

**Authors:** Darién Ledesma, Eduardo Berriatua, M. Carmen Thomas, Luis Jesús Bernal, María Ortuño, Celia Benitez, Adriana Egui, Kostas Papasouliotis, Bryn Tennant, Julia Chambers, Juan José Infante, Manuel Carlos López

**Affiliations:** 1 Instituto de Parasitología y Biomedicina “López Neyra” Consejo Superior de Investigaciones Científicas, Granada, Spain; 2 Departamento de Sanidad Animal, Universidad de Murcia, Murcia, Spain; 3 Departamento de Medicina y Cirugía Animal, Universidad de Murcia, Murcia, Spain; 4 School of Veterinary Sciences, University of Bristol, Bristol, UK; 5 Capital Diagnostics, SAC Consulting, Penicuik, Midlothian, UK; 6 Animal and Veterinary Sciences, Roslin Institute, Easter Bush, Midlothian, UK; 7 Bioorganic Research and Services, S.A. (Bionaturis Group) Jerez de la Frontera, Jerez de la Frontera, Spain

**Keywords:** Canine, Leishmania, PFR1 recombinant antigen, Serological, Diagnosis

## Abstract

**Background:**

*Leishmania infantum* is a protozoan parasite transmitted by phlebotomine sand flies that causes life-threatening disease in humans and dogs. The dog is the primary reservoir of the parasite and early diagnosis of canine leishmaniosis is crucial at the clinical and epidemiological level. The currently available serological tests for CanL diagnostic show limitations therefore the aim of the present study was to investigate the diagnostic performance of an indirect antibody ELISA based on the *Leishmania infantum* recombinant antigen PFR1 in asymptomatically infected dogs. One hundred fifty-six dogs including *Leishmania*-free experimental Beagles and pet dogs from England, Scotland and Leishmania-endemic Murcia in Spain, were tested with the assay. The later were also tested with two commercial *L. infantum* crude antigen ELISAs (INgezim and Civtest, respectively) and a real-time kinetoplast PCR test.

**Results:**

Anti-PFR1 antibodies were detected in the four groups of dogs, and the mean log-transformed optical density (OD) values were lowest in Beagles and in dogs from England and highest among dogs from Murcia (*p* < 0.05). Using the highest OD in beagles as the PFR1 ELISA cut-off point, the estimated seroprevalence was 27% (14-40%) in dogs from Murcia, 4% (0-9%) in dogs from Scotland and 3% (0-8%) in dogs from England (*p* < 0.05). Seroprevalence in dogs from Murcia according to the INgezim and Civtest ELISAs were 24% (12-37%) and 31% (18-45%), respectively, whilst the prevalence of infection based on PCR in these dogs was 73% (60-86). The percentages of PFR1-positive dogs that tested negative on the INgezim and Civtest ELISAs were 30% and 35%, respectively, and all of them tested positive on the PCR test. Relative to the PCR, the specificity, sensitivity and area under the ROC curve of the PFR1 ELISA were 100%, 36% and 0.74 (0.63-0.86), respectively.

**Conclusions:**

The ability shown by the PFR1 ELISA to detect infected dogs that go undetected by the crude antigen ELISAs is clinically and epidemiologically useful and PFR1 could be considered a candidate for a multi-antigen-based immunoassay for early detection of *L. infantum* infected dogs.

**Electronic supplementary material:**

The online version of this article (10.1186/s12917-017-1224-z) contains supplementary material, which is available to authorized users.

## Background

*Leishmania infantum* (*L. infantum*) transmitted by phlebotomine sand flies, infects reticuloendothelial cells causing potentially life-threatening human and canine leishmaniosis (CanL). Since the advent of PCR diagnosis, it has been found that 50-80% of dogs endemic to areas such as the Murcia Region in Southeast Spain are chronically infected and that most remain asymptomatic [1]. The development of the disease is strongly influenced by the host’s immunity, which tends to be polarized towards either a Th1 cell- or a Th2 antibody-mediated response [2, 3]. The latter is commonly associated with disease susceptibility resulting from the widespread deposition of antibody-antigen complexes in capillaries. Subclinically infected dogs, particularly those in preclinical stages, may transmit infection to sand flies [4, 5]. Consequently, serological diagnosis of *Leishmania* infection is clinically and epidemiologically useful.

The estimated seroprevalence of CanL in endemic areas is typically 10-30% depending on the dog’s habitat and exposure to infection as well as on the sensitivity (Se) and specificity (Sp) of the diagnostic test. Se and Sp vary according to the antigens used - crude, soluble, purified or recombinant antigens - and the immunological method used for detection. The most common types of immunoassay used for epidemiological and surveillance purposes are indirect immunofluorescence assays (IFAs), enzyme-linked immunosorbent assays (ELISAs), and rapid immunochromatographic tests (ICTs) [6, 7]. The IFA is considered the reference test. However, its Se may range from 60% to 100% [8, 9], and it is known that false positives may arise from cross-reactivity with other protozoan and bacterial infections [7, 10, 11]. The antigens used in these types of tests might be either crude antigens obtained from cultures of the parasite or recombinant antigens expressed in heterologous expression systems. The tests based on recombinant antigens are more specific and easier to produce and standardize and have been used for *Leishmania* spp. serodiagnosis in human and dogs [12–15]. Among the antigens composing these tests, rK39, a repetitive, conserved, protein in *Leishmania donovani* complex species [16], has been widely used. The validity of tests based on rK39 depends on time since infection and presence of active disease. In a recent meta-analysis study, the overall Se of rK39-based ICT tests in infected asymptomatic dogs was only 50% [17]. The authors suggested that using a combination of recombinant antigens should improve test Se. The advantage of this approach in the serological diagnosis of *L. infantum* infection in dogs was later shown [14].

In the search for vaccine candidates, a highly immunogenic protein from *L. infantum* named PFR1 was cloned and expressed as recombinant protein. PFRs, or paraflagellar rod proteins, represent a family of relevant trypanosomatid antigens located in the paraflagellar pocket of these parasites [18–20]. Knockout assays in *Leishmania mexicana* evidenced that the proteins encoded by PFR genes play a critical role in the mobility and survival of the parasite [21]. Some members of the PFR antigen family stand out for their high immunogenicity [22]. The sera from both asymptomatic and cardiac Chagas’ disease patients showed a higher level of antibodies against PFR antigens of *Trypanosoma cruzi* (*T. cruzi*) than sera from healthy donors [23].

Given the limitations of presently available serological tests for CanL and the high immunogenicity of PFR antigens, the present article evaluated the seroreactivity of dogs to the PFR1 recombinant protein and the validity of a PFR1-based ELISA for the diagnosis of asymptomatic CanL.

## Methods

### Cloning of PFR1 coding sequence in pQE_32_ expression vector; overexpression and purification of the PFR1 recombinant antigen

The PFR1 coding sequence was amplified by PCR using *L. infantum* genomic DNA as a template along with the primers PFR1Li-ATG (5’GAATGGATCCCCCCTGAAGATGCG3’) and PFR1Li-TAAKpn1 (5’GTAAGGTACCCCTCCAGCTGCGTGCTCG3’), which bear *Bam*HI and *Kpn*I restriction sites, respectively. The 1775 bp amplified fragment was cloned into a pGEM-T Easy vector (Promega®) and sequenced. The PFR1 gene was subsequently excised from the pGEM-T-PFR1 clone by *Bam*HI and *Asp*718 digestion and in-frame cloned into the *Escherichia coli* (*E. coli*) pQE_32_ expression vector digested with the same enzymes. The resulting clone was sequenced and named pQE-32 PFR1Li.

**Fig. 1:**
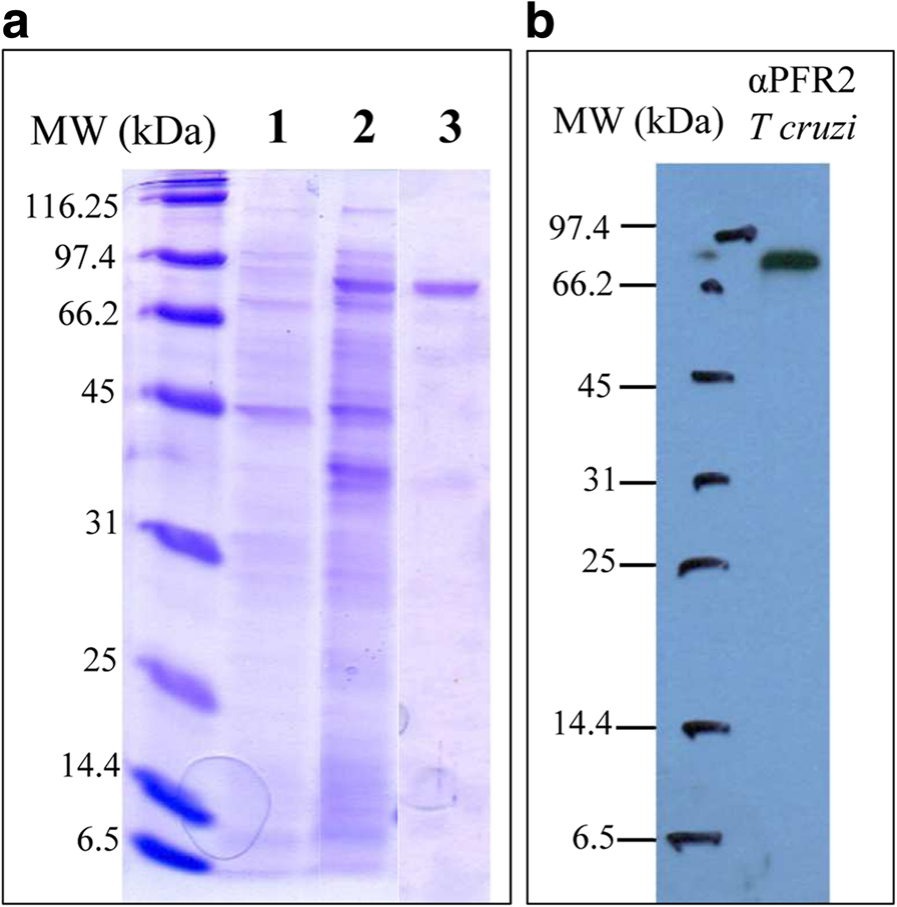
Expression and purification of *L. infantum* recombinant PFR1 protein. **a** Analysis by SDS-PAGE and Coomassie blue staining of the protein purification process. The *Escherichia coli* M15 strain was chosen as the host bacterium for *Li*PFR1 overexpression (lane 1). An intense band of approximately 70 kDa was observed after IPTG induction (lane 2) and not seen in uninduced cultures (lane 1). Purified *Li*PFR1 protein after purification by Ni^2+^ affinity chromatography is shown in lane 3. MW, molecular weight marker (kDa). **b** Western blot analysis of *L. infantum* PFR1 recombinant protein by using α-PFR2 antibody against the homologous PFR2 protein from *T. cruzi*. MW, molecular weight marker (kDa)

The recombinant PFR1 protein was overexpressed by adding 0.02 mM of isopropyl-beta-D-thiogalactopyranoside (IPTG) to the *E. coli* M15 strain transformed with pQE-32-PFR1Li and grown for 3 h at 37 °C. Total proteins were solubilized in solubilization buffer (0.3 M NaCl, 50 mM NaH_2_PO_4_, 1 mM phenyl methyl sulfonyl fluoride, pH 8.0) and by sonication. Recombinant PFR1 was subsequently purified to homogeneity under native conditions by Ni^2+^-NTA affinity chromatography to a 6× histidine tag placed at the NH_2_-terminus of PFR1. After washing, the protein was eluted in solubilization buffer at pH 6.0. The final elution fraction resulting from the purification process was analysed in triplicate by 10% SDS-PAGE and Coomassie Blue staining (Fig. 1). The protein concentration was measured with a Micro BCA Protein Assay Kit (Thermo).

### Western blot analysis

Purified PFR1 recombinant protein was electrophoresed in a 10% SDS-PAGE and transferred to PVDF membranes (Immobilon-P) by following standard procedures as previously described [24]. Western blotting was performed in duplicate according to standard techniques. For PFR1 detection, an antibody raised against the PFR2 protein of *T. cruzi* was used as primary antibody [20] at 1:2000 dilution. The secondary antibody was an HRP-conjugated anti-rabbit IgG (Sigma) at 1:20,000 dilution. The blots were subjected to peroxidase and luminol/enhancer solutions using a SuperSignal® West Pico Chemiluminescent Reagent Kit (Thermo Scientific), and subsequently exposed to Kodak X-Omat autoradiographic film.

### Study population and design

The study was performed using samples from 156 dogs (58% males and 42% females), including 70 dogs from Murcia participating in Leishmania research projects at the University of Murcia and 86 dogs from the United Kingdom undergoing veterinary care for problems unrelated to *Leishmania* infection. Murcia samples came from two groups of dogs: 25 beagles and 45 mixed-breed dogs aged between 6 months and 15 years old (Beagles; mean 1 year and 6 months. Mixed-breed Murcia group; mean 3 years and 7 months). Similarly, samples from UK dogs were from 50 dogs aged between 3 months and-15 years old (mean 7 years and 4 months) coming from Scotland and 36 dogs aged between 4 months and 13 years old (mean 6 years and 8 months) coming from South West England. All dogs from the UK were cross-breed.

The beagles had been recently bought from an authorized breeder (Isoquimen SL., Barcelona, Spain) for a *L. infantum* vaccination trial, who selected the dogs based on age and gender balance. They were used as a *Leishmania*-negative control group after their status was confirmed by an IFAT serological test performed by the breeder and a real-time *Leishmania* kinetoplast-specific PCR (kPCR) using DNA from bone marrow samples as a template (described below). The Murcia group of dogs were a random selection of mostly abandoned animals living in peri-urban areas of the city of Murcia, a typically *L. infantum* endemic area [25], rescued and euthanized by the local authority as part of a municipal zoonosis control program. Serum and tissue (spleen, lymph node and skin) samples taken immediately after death were similarly tested for *L. infantum* antibodies using two commercial ELISA tests (described below) and the kPCR, respectively. The UK dogs were pet animals and samples used was surplus serum taken for other diagnostic tests. Hence, they were not tested for *L. infantum* DNA and were only analysed for *L. infantum* antibodies with the PFR1 ELISA assay developed in this work (described below). The UK is a *Leishmania*-free country. However, the travel history of the UK dogs incorporated in this study was unknown. We cannot rule out that some of the UK dogs had been in *Leishmania*-endemic Southern Europe and exposed to *Leishmania* infection.

### Detection of specific anti-PFR1 antibodies by ELISA

Serum from all dogs, obtained from blood samples collected from the cephalic vein into vacuum tubes, was tested for anti-PFR1 antibodies by ELISA. ELISAs were performed in triplicate at different dilutions by following previously described procedures [26]. Positive and negative control sera were included in all plates. Briefly, ELISA 8-well strips (Nunc-Immuno module F16; Roskilde, Denmark) were coated with 0.5 μg/well of PFR1 in a carbonate buffer. After being coated, the plates were stored in a dry atmosphere at −20 °C until use. The wells were washed twice with 200 μL of PBS-0.05% TWEEN 20 and blocked by incubation for 90 min with 5% non-fat dried milk powder in PBS (blocking solution) at 37 °C. Subsequently, canine sera at 1/100 and 1/200 dilutions in blocking solution were added to the dry blocked wells and incubated for 2 h at 37 °C. Before the secondary antibody was added, the plates were washed five times with 200 μL of PBS-0.05% TWEEN 20 and incubated for 1 h with peroxidase-conjugated anti-dog IgG (Sigma) in blocking solution at a 1/10000 dilution at 37 °C. After incubation, the plates were washed five times with 200 μL of PBS-0.05% TWEEN 20, and the reaction was developed using ortho-phenylenediamine and hydrogen peroxide in a citrate buffer for 5 min in the dark at room temperature. Finally, 8 N sulfuric acid was used to stop the reaction, and the absorbance was measured at 492 nm.

### Commercial *L. infantum* antibody ELISAs

Commercial antibody tests to detect serum *L. infantum* IgG included INgezim Leishmania® (Ingenasa, Spain) and Civtest Canis Leishmania® (Hipra, Spain, later commercialized by Esteve, Spain as Leiscan®). Both tests use crude immunodominant *L. infantum* antigens for capturing specific IgGs. For detection, the INgezim test uses conjugation to a specific canine IgG monoclonal antibody, whilst Civtest uses a generic protein A/HRPO conjugate. The validity of these tests has been assessed by the manufacturers using IFA as the reference test. The INgezim results showed 95% and 80% agreement for 1/100 and 1/160 IFA cut-offs, respectively, whilst the estimated sensitivity (Se) and specificity (Sp) of the Civtest assay were 98% and 96%, respectively. Moreover, the performance of both tests was recently assessed on experimentally infected dogs [27], and Sp was 100% for both tests whilst Se was 98% for Civtest and 78% for INgezim.

Samples were done in duplicate, and antibody optical densities (OD) were read in a spectrophotometer. The mean of the two readings was used to classify samples as positive, negative, or inconclusive by following the manufacturers’ instructions.

### *L. infantum*-specific real-time PCR

Tissue samples from dogs of the Murcia group were stored at −20 °C until used for DNA purification using a nucleic acid purification robot (Maxwell® 16, Promega). Bone-marrow samples of Beagles were obtained by needle aspiration, mixed with a protein digestion solution (10 mM Tris-HCl, 10 mM NaCl, 10 mM EDTA, pH = 8; 0.1 mg/ml of proteinase K and 1% of SDS) and the DNA was extracted within 48 h with DNeasy Blood and tissue kit, Quiagen.. The DNA was analysed for *L. infantum* kinetoplast sequences using a TaqMan probe in a real-time PCR [28, 29]. The analyses were done in duplicate using 300 ng of high-quality template DNA (A260/A280 ≥ 1.7) per PCR reaction. Samples from dogs with clinical leishmaniosis and uninfected dogs were used as positive and negative amplification controls, respectively. A semi-quantitative measure of parasite DNA load was obtained by estimating the PCR amplification threshold cycle (CT) at which near-logarithmic PCR product generation was detected [30]. Samples with CT = 1-38 were considered positive.

### Statistical analysis

Analyses were carried out in R (http://www.R-project.org). Anti-PFR1 antibody OD distributions were analysed and normalized using a decimal logarithmic transformation of ODx100 (LOD). The mean LODs between levels of explanatory variables, including dog origin, gender and age (categorized as ≤1 yrs., 2 yrs., 3 yrs. and ≥4 yrs), were compared using ANOVA. A multivariable linear regression model was then used to investigate the independent contributions of explanatory variables to LOD in dogs of the Murcia group [31].

A LOD cut-off value was selected to classify dogs as seronegative or seropositive by adding one decimal to the largest OD among the *Leishmania*-negative Beagle dogs. Differences in the proportion of seropositive dogs across levels of explanatory variables were analysed using the chi-squared test or with Fisher’s exact test when one of the expected values in the contingency table was less than five [32].

A logistic regression model was developed to analyse the multivariable relationship between the serological status of the Murcia dogs and their age and gender.

Receiver operating characteristic (ROC) curves [33] were used to evaluate the performance of the test and calculate Se, Sp with respect to the PCR test in skin and lymphoid tissue samples, considered the reference test [34].

Cohen’s kappa coefficient [32] was employed to evaluate the degree of agreement between the results of qualitative tests by following the scale: 0, no agreement; >0- < 0.2, slight; 0.2- < 0.4, fair; 0.4- < 0.6, moderate; 0.6- < 0.8, substantial; and >0.8, almost perfect.

A 5% (*p* < 0.05) significance level for a two-tailed test was considered in all comparisons.

## Results

### Purification of *Leishmania infantum* recombinant PFR1 protein

Figure 1a shows the analysis by SDS-PAGE of the PFR1 recombinant protein purified as described in Materials and Methods. An intensely stained band with the expected electrophoretic mobility of approximately 70 kDa was observed (Fig. 1a, lane 3). The purity was >95% as assessed by band densitometry analysis after Coomassie blue staining.

The 70 kDa band was the only one detected by the antibody used in the Western blot (Fig. 1b), therefore indicating the integrity of the purified PFR1 protein. In addition, the purified protein was tested with the E-Toxate reaction kit (Sigma), which showed it to be free from bacterial LPS contaminants.

### Antibody optical density distribution for PFR1 ELISA

The mean and median (range) LOD values obtained in the PFR1 ELISA were 0.705 and 0.680 (0.014-1.760), respectively, and differed significantly according to the dogs’ origin (Table 1). Median LODs were lower for beagles and dogs from England than for the Murcia dogs (*p* < 0.05). The remaining differences in median LOD between groups were not statistically significant (*p* < 0.05) (Table 1).

**Table 1:** PFR1 normalized optical density according to origin, age and gender

Variable	Level	N° dogs	Mean	Percentiles						
				0%	10%	25%	50%	75%	90%	100%
Origin	Beagles	25	0.651	0.067	0.241	0.335	0.490	0.673	0.899	1.079
	Murcia	45	0.706	0.247	0.563	0.653	0.746	1.160	1.245	1.760
	England	36	0.844	0.014	0.301	0.546	0.645	0.720	1.021	1.346
	Scotland	50	0.532	0.091	0.322	0.443	0.734	0.957	1.057	1.509
	All	156	0.705	0.014	0.319	0.510	0.680	0.892	1.076	1.760
Age (years)^a^	≤1	8	0.686	0.247	0.280	0.488	0.655	0.824	1.190	1.193
	2	15	0.757	0.426	0.500	0.625	0.746	0.814	1.066	1.239
	3	8	0.800	0.580	0.663	0.712	0.731	0.792	1.031	1.272
	4	12	1.057	0.630	0.644	0.740	1.020	1.256	1.559	1.760
Gender^a^	Male	24	0.772	0.426	0.560	0.638	0.716	0.856	1.203	1.249
	Female	19	0.978	0.294	0.634	0.724	0.845	1.216	1.340	1.760

^a^Dogs from Murcia only

Within the Murcia group, LOD was positively associated with age and the median LOD was greater in females than in males (Table 1). The multivariable linear regression model confirmed the independent association of both age and gender with LOD (results not tabulated) (*p* < 0.05).

### Estimated seroprevalence based on the PFR1 and commercial ELISAs. Prevalence based on PCR and diagnostic performance of the PFR1 ELISA

Considering a cut-off LOD of 1.080 - a value one thousandth of a unit higher than the highest LOD in the beagles (Table 1) - PFR1 seroprevalence (95% CI) was 27% (14-40) in dogs from Murcia, 4% (0-9) in dogs from Scotland and 3% (0-8) in dogs from England (*p* < 0.05) (Table 2).

**Table 2:** Estimated PFR1, Ingezym and Civtest ELISA seroprevalence according to dog origin, age and gender

Variable	Level	N° dogs	PFR1	Ingezym	Civtest	PCR
Origen	Murcia	45	27 (14–40)	24 (12–37)	31 (18–45)	73 (60–86)
	Beagles	25	0 (0–0)	–	–	0 (0–0)
	Scotland	50	4 (0–9)	–	–	–
	England	36	3 (0–8)	–	–	–
Age (years)^a^	≤1	8	25 (0–55)	0 (0–0)	13 (0–35)	75 (45–100)
	2	15	13 (0–31)	27 (4–49)	40 (15–65)	93 (81–100)
	3	8	13 (0–35)	38 (4–71)	25 (0–55)	50 (15–85)
	≥4	12	50 (22–78)	25 (1–50)	33 (7–60)	67 (40–93)
Gender^a^	Male	24	17 (2–32)	21 (5–37)	25 (8–42)	71 (53–89)
	Female	19	42 (20–64)	32 (11–52)	42 (20–64)	79 (61–97)

^a^Dogs from Murcia only

The estimated seroprevalence in dogs from Murcia, according to the INgezim and Civtest ELISAs, was 24% (12-37) and 31% (18-45), respectively. The prevalence of asymptomatic infection assessed by PCR in these dogs was 73% (60-86) (Table 2).

In logistic regression models including gender and age, the probability of being seropositive in dogs from Murcia was not significantly associated to either variable.

Relative to the PCR test, which was performed only in the beagles and Murcia dogs, the PFR1 ELISA Sp and Se at a cut-off of 1.080 were 100% and 36%, respectively. The AUC (95% CI) was 0.74 (0.63-0.86).

### Diagnostic agreement between tests

The levels of agreement between the different diagnostic tests performed in dogs from Murcia are shown in Table 3. The kappa coefficient from comparing the PFR1 ELISA with other serological techniques ranged from 0.23 to 0.36 (fair agreement), while the result of comparing the commercial ELISAs INgezim and Civtest was 0.61 (substantial agreement). Comparing the serological tests with the PCR test, the kappa coefficient ranged from 0.14 (slight agreement) for INgezim to 0.28 (fair agreement) for Civtest (Table 3).

**Table 3:** Kappa coefficient and degree of agreement between PFR1, INgezim (Ing) and Civtest (Civ) ELISA tests

Techniques	N° dogs	PFR1	Ing	Civ	Ing + Civ	PCR
PFR1	45	–	Fair	Fair	Fair	Fair
Ing	45	0.36 (0.05–0.67)	–	Substantial	Substantial	Slight
Civ	45	0.24 (0.00–0.55)	0.61 (0.36–0.87)	–	Almost perfect	Fair
Ing + Civ	45	0.28 (0.00–0.57)	0.74 (0.53–0.95)	0.90 (0.77–1.00)		
PCR	45	0.23 (0.08–0.39)	0.14 (0.00–0.29)	0.28 (0.11–0.45)	0.26 (0.06–0.45)	–

Table 4 shows the percentage of discordant samples tested with two serological tests (T1 and T2) and the percentage of these samples that were PCR positive. The percentage of PFR1 ELISA positives that were negative to the INgezim and Civtest ELISAs were 35% and 30%, respectively, and all these samples were PCR positive. On the other hand, the percentage of samples that tested negative on the PFR1 ELISA and positive on the INgezim and Civtest ELISAs were 29% and 40%, respectively, and whilst all of the latter (Civtest ELISAs) were PCR positive, only 80% of the former (INgezim) were PCR positive (Table 4).

**Table 4:** Percentage of positives to one of two ELISA techniques (T1 and T2) including PFR1, INgezim (Ing) and Civtest (Civ) ELISA tests, and their PCR status

Techniques		T1+; T2-		T1-; T2+	
T1	T2	% Seropositive	% PCR positive	% Seropositive	% PCR positive
PFR1	Ing	30	100	29	80
PFR1	Civ	35	100	40	100
PFR1	Ing + Civ	24	100	43	100
Ing	Civ	14	50	31	100
Ing	Ing + Civ	0	–	31	100
Civ	Ing + Civ	0	–	13	50

## Discussion

The results obtained in the present study are consistent with PFR1 stimulating a specific antibody response in dogs asymptomatically infected with *L. infantum*. One-third of all seropositive dogs were only detected with the PFR1 ELISA and not by the commercial ELISAs. Conversely, the PFR1 ELISA failed to detect another third of seropositive dogs according to the commercial ELISAs. Together, these results suggest that the PFR1 antigen might be a useful candidate to incorporate in a multi-antigen ELISA for serological detection of asymptomatically infected dogs. To further explore this use, cross-reactivity between PFR1 and other recombinant antigens used for *Leishmania* infection diagnosis such as the rK39 antigen should be analysed.

Although the UK is a *Leishmania*-free zone, 3-4% of the UK dogs included in this study tested positive to the PFR1 ELISA. This result might indicate that some PFR1 conformational epitopes are shared with proteins present in other pathogens or that the seropositive UK dogs were actually infected with *L. infantum*. The latter cannot be ruled out since the travel history of the UK dogs was unknown and no samples were available for PCR testing. Serological cross-reaction has been reported between *Leishmania* antigens and antigens of other pathogens present in the UK such as *Ehrlichia canis*, *Babesia canis*, *Anaplasma phagocytophilum*, *Rickettsia conori*, *Toxoplasma gondii, Neospora caninum* and *Hepatozoon canis* [7, 10, 11, 35]. However, arguing against cross-reactivity, at the selected cut-off LOD of 1.080, the specificity of the PFR1 ELISA relative to the PCR test performed only in beagles and dogs from Murcia was 100%. In comparison, the homologous PFR2 protein from *T. cruzi* is recognized by the sera from asymptomatic chagasic patients with a specificity and sensitivity of 92% and 75%, respectively. It is worth mentioning that the sera from patients with leishmaniasis also recognize this protein.

The sensitivity of the PFR1 ELISA compared to the PCR test was 36%, similar to that estimated for commercial ELISAs. This low sensitivity value might be expected when analysing asymptomatically infected dogs since most dogs develop a predominantly cellular immune response to the parasite while the humoural response might be variable and in some cases undetectable by these tests [34]. The reason why the PFR1 ELISA identified specific antibodies that were not detected by the commercial tests based on crude immunodominant antigens is unclear. In crude antigen extract preparations, specific epitopes might not be available for binding specific antibodies.

PFR1 ELISA LODs and seroprevalence were greater in older dogs and LODs in females compared to males. Given the small number of dogs from Murcia, the accuracy of age and gender-specific estimates in this study was limited. There is no previous evidence of gender-specific susceptibility to leishmaniasis, but increasing seroprevalence during the first years of life is typical and is associated with accumulated exposure to infection [36]. Age- and sex-specific differences between studies depend on the degree to which dogs are naturally exposed to *Leishmania* infection, which is greatest for dogs living outdoors in peri-urban and rural areas and not receiving preventive insecticidal treatments [25].

In summary, the present study highlights the potential of PFR1 recombinant antigen for *L. infantum* serological diagnoses. Further studies need to be carried out to investigate the test specificity and its performance when combined with other relevant recombinant antigens for diagnostics of CanL.

## Conclusions

PFR1 recombinant antigen detects specific antibody responses in *L. infantum*-infected dogs and could be a useful antigen to incorporate in a multi-antigen immunoassay to detect asymptomatically infected dogs.

## Additional file

Additional file 1: Study data. (XLSX 140 kb)

## Abbreviations

bp: base pairs
CanL: Canine leishmaniosis
Civ: Civtest
*E. coli*: *Escherichia coli*
ELISA: Enzyme-linked immunosorbent assay
ICT: Immunochromatographic test
IFAT: Indirect immunofluorescence assay
IgG: Immunoglobulin G
Ing: INgezim
IPTG: Isopropyl-beta-D-thiogalactopyranoside
kPCR: kinetoplast-specific PCR
*L. infantum*: *Leishmania infantum*
LOD: normalised, log-transformed OD
OD: Antibody optical density
PBS: Phosphate buffered saline
PCR: Polymerase chain reaction
PVDF: Polyvinylidene difluoride
ROC: Receiver operating characteristic
SDS-PAGE: Sodium dodecyl sulfate polyacrylamide gel electrophoresis
Se: Sensitivity
Sp: Specificity
*T. cruzi*: *Trypanosoma cruzi*
TC: Threshold cycle
Th cells: T lymphocyte helper cells
UK: United Kingdom
